# Increased Serum Soluble Urokinase Plasminogen Activator Receptor Predicts Short-Term Outcome in Patients with Hepatitis B-Related Acute-on-Chronic Liver Failure

**DOI:** 10.1155/2019/3467690

**Published:** 2019-05-02

**Authors:** Zuxiong Huang, Ning Wang, Shuiwen Huang, Yi Chen, Shida Yang, Qiaorong Gan, Hanhui Ye, Baorong Liu, Chen Pan

**Affiliations:** ^1^Department of Hepatology, Mengchao Hepatobiliary Hospital of Fujian Medical University, Fuzhou, Fujian Province, China; ^2^Department of Hepatology, Affiliated Infectious Disease Hospital of Fujian Medical University, Fuzhou, Fujian Province, China; ^3^Department of Infectious Disease, The First People's Hospital of Fuzhou, Jianxi Province, China

## Abstract

**Aims:**

Soluble urokinase plasminogen activator receptor (suPAR) reflects the immune activation in circumstances of inflammation and infection. It has been considered as a risk biomarker associated with poor outcome in various low-grade inflammation and infectious diseases. The study is aimed at investigating whether suPAR has a predictive value with short-term survival in patients with hepatitis B-related acute-on-chronic liver failure (HB-ACLF).

**Methods:**

Serum suPAR expression was compared among patients with different states of chronic hepatitis B virus infection. Sixty HB-ACLF patients were recruited as the training cohort and followed up for 90 days. Serum suPAR level and the clinical relevance with short-term outcome were investigated. The temporal dynamics of suPAR were evaluated in 50 HB-ACLF patients with available serum sequentially at baseline, week 2 and week 4. Another 167 HB-ACLF patients were enrolled to validate the predictive value of suPAR with respect to the prognosis.

**Results:**

Serum suPAR level was significantly increased in HB-ACLF patients compared to non-ACLF patients. In the training set of HB-ACLF, we observed higher suPAR level, INR, MELD score, and more complications in nonsurvivors than survivors. Longitudinal analysis revealed an increased trend of suPAR level in nonsurvivors during week 0 to week 4 and the modest decline in survivors. It showed that the synchronous suPAR level was higher in nonsurvivors at all indicated time points. Elevated suPAR level at baseline was identified as a strong predictor of a 90-day mortality of HB-ACLF patients. It was confirmed suPAR > 16.26 ng/ml had a positive predictive value of 72.22% and a negative predictive value of 77.88% for poor outcome in the validation cohort.

**Conclusions:**

Serum suPAR level increases significantly in HB-ACLF patients and associated with a 90-day mortality. It suggests that suPAR might be a potential biomarker to predict the prognosis of HB-ACLF patients.

## 1. Introduction

Acute-on-chronic liver failure (ACLF) is a syndrome characterized by acute decompensation of chronic liver disease associated with organ failures and high short-term mortality [1]. Recently, systemic inflammation (SI) has been considered as the primary driver involved in ACLF [2–4]. Systemic inflammation hypothesis was proposed on the basis of the CANONIC study. It was observed that nonspecific SI markers such as white cell count (WCC), the serum levels of C-reactive protein (CRP), major cytokines (i.e., interleukin- (IL-) 6, tumor necrosis factor- (TNF-) *α*), and chemokines (e.g., IL-8) were remarkably elevated in ACLF patients, paralleling with the severity of the syndrome [2]. SI might be due to the translocation of proinflammatory molecules (pathogen-associated molecular patterns, PAMPs) from the intestinal lumen to the systemic circulation and/or to the release or damage-associated molecular patterns (DAMPs) from the diseased liver or another organ. Precipitating events (active alcoholism/acute alcoholic hepatitis, bacterial infections, and others) might induce an acute exacerbation of SI and promote the development of ACLF. Severity of SI at enrolment and progression or regression of SI during hospitalization is closely associated with short-term prognosis [4, 5]. It is valuable to find some specific and sensitive biomarkers of systemic inflammation, beneficial to early identify and initiate treatment in ACLF. Until now, the optimal indicator of systemic inflammation is still scarce and it is a work in progress [5].

The urokinase plasminogen activator receptor (uPAR) is expressed on most leucocytes including neutrophils, lymphocytes, monocytes, and macrophages. The uPAR can be cleaved from the cell surface in circumstances of inflammation and infection, and then the soluble form of the receptor (suPAR) is formatted. As a result of its broad expression and release from many activated leucocytes, suPAR has been suggested as a highly sensitive biomarker for mirroring the degree of immune activation. High serum suPAR concentration has been associated with increased mortality in systemic inflammation response syndrome, sepsis, bacteremia, and critically ill patients [6–9]. Interestingly, the predictive potential of suPAR is superior to that of commonly used inflammatory markers such as CRP and procalcitonin (PCT) level [6, 10]. Recently, it has been found that circulating suPAR level was evelated in patients with chronic liver disease, acute liver failure (ALF), and decompensated cirrhosis [11–13]. However, the expression and dynamic process of suPAR in patients with hepatitis B-related ACLF (HB-ACLF) are less defined. Whether suPAR is a promising biomarker associated with the prognosis of HB-ACLF patients needs to clarify.

In this study, we enrolled a prospective clinical cohort to investigate the clinical relevance of serum suPAR level with the short-term prognosis and performed a dynamic longitudinal observation of suPAR expression in patients with HB-ACLF. We also assess another large real-life cohort of HB-ACLF patients to validate the predictive accuracy and reliability of suPAR with respect to the outcome.

## 2. Patients and Methods

### 2.1. Patients

A total of 60 patients with HB-ACLF admitted to Mengchao Hepatobiliary Hospital of Fujian Medical University (Fuzhou, China) from January 2012 through December 2013 were included as the training set. All patients had available blood samples at enrollment and were followed until either their death or the end of the 90-day follow-up period. Among them, 50 patients received the prospective intensive management and the blood samples were regularly collected at baseline, week 2 and week 4 during hospitalization. In addition, chronic hepatitis B patients (CHB, *n* = 38) and HBV carriers in immune tolerance phase (IT, *n* = 33) were enrolled as disease controls. Healthy controls (HC, *n* = 33) were also recruited to compare serum suPAR level in the same period. As an initial exploration, the association between suPAR at enrollment with 90-day outcome was investigated in the training set of ACLF. The dynamic changes of suPAR were also observed regularly during the hospitalization. Another 167 consecutive patients with HB-ACLF admitted to the same tertiary hospital from January 2014 through December 2016 were included as a real-life cohort to validate the predictive value of serum suPAR. All patients were required to undergo a review of their medical history and a physical examination. This study was conformed to the ethical guidelines of the 1975 Declaration of Helsinki. Approval of this study was obtained from the Ethics Committee of Mengchao Hepatobiliary Hospital of Fujian Medical University, and informed consent was obtained from each patient or his/her legal guardian.

### 2.2. APASL Diagnostic Criteria for Hepatitis B-Related ACLF

HB-ACLF was diagnosed according to consensus recommendations of the Asian Pacific Association for the Study of the Liver in 2014 [14]. The inclusion criteria was defined as follows: (1) a history of chronic hepatitis with HBsAg positive for at least the previous 6 months and (2) progressive jaundice with serum bilirubin ≥ 5 mg/dl (85 *μ*mol/l) and coagulopathy with INR ≥ 1.5 or prothrombin activity < 40% complicated within 4 weeks by clinical ascites and/or encephalopathy. The exclusion criteria were as follows: (1) hepatitis C or D or human immunodeficiency virus coinfection, and liver disease because of other etiology, (2) use of hepatotoxic drugs or regular alcohol consumption, (3) evidence of hepatocellular carcinoma, and (4) previous liver or kidney transplantation.

### 2.3. Data Collection

Clinical and laboratory data were collected at the time of admission including age, gender, HBeAg status, HBV-DNA load, serum bilirubin, albumin, ALT, AST, INR, WCC, platelet counts, and serum creatinine. Complications were also taken into account and recorded as follows: clinical proved infection, gastrointestinal bleeding (GB), hepatic encephalopathy (HE), and hepatorenal syndrome (HRS). Lithium heparin plasma samples were obtained for testing CRP and PCT immediately after blood collection on a fully automated laboratory analyzer (Cobas 8000; Roche Diagnostics, Rotkreuz, Switzerland). Severity of liver disease was assessed by model for end-stage liver disease (MELD) and chronic liver failure consortium organ failure (CLIF-C OF) score for each patient.

### 2.4. Measurement of suPAR

All blood samples of patients with the HB-ACLF, CHB, IT, and HC groups at enrollment were obtained. Sequential blood samples at prespecified visits (baseline, 2 weeks and 4 weeks after enrolment) were collected in 50 HB-ACLF patients with prospective intensive surveillance during hospitalization. All samples were obtained in EDTA tubes, centrifuged at approximately 1500 ×g for 15 min, and store frozen at -80°C until analysis. Serum suPAR level was determined using the enzyme-linked immunosorbent assay (ELISA) method according to the manufacturer's protocols (GeneTex, Taiwan). All samples were tested in duplicate.

### 2.5. Statistical Analysis

Data were given as median and range due to the skewed distribution of most of the parameters. Counts and percentages were used for the description of the categorical variables. Comparisons between two independent groups were made with the Mann-Whitney *U* test. Multiple comparisons more than two groups were conducted by the Kruskal-Wallis ANOVA and Mann-Whitney *U* test for post hoc analysis. For categorical variables, comparisons among groups were made with the Chi-squared tests or Fisher test if appropriate. To explore the predictive value of different prognostic scoring systems, area under the receiver operating curve (ROC) was calculated and compared by the *Z* test. The optimal cut-off value was identified based on a maximum sum of sensitivity and specificity for the prediction of survival in HB-ACLF patients. Three-month survival probability curves were calculated with the Kaplan-Meier method and compared with the log-rank test. The multivariate logistic regression models were fitted to select the main factors independently associated with the end point. A forward stepwise procedure (variable entry/drop criteria, *p* < 0.05/*p* > 0.2) was applied to select factors significantly contributing to the model fit. Results of the multivariate analysis were presented as *p* values, odds ratio, and 95% confidence interval. The significance level for all statistical tests was set at 0.05 two-tailed. All statistical analyses were performed using SPSS 18.0 and figures were drawn using GraphPad Prism 6.

## 3. Results

### 3.1. Serum suPAR Level Is Significantly Increased in HB-ACLF Patients

**Figure 1:**
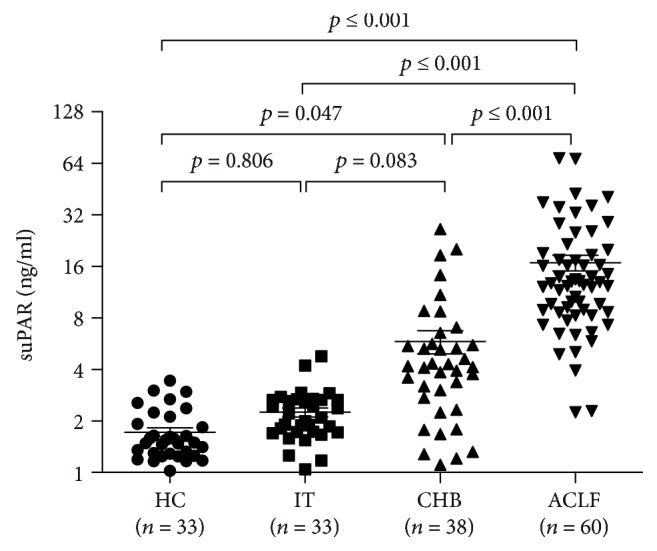
Serum suPAR concentration was increased in patients with hepatitis B-related acute-on-chronic liver failure. The comparison of serum suPAR concentration in the health controls (HC), HBV carriers in immune tolerance (IT), chronic hepatitis B (CHB), and hepatitis B-related acute-on-chronic liver failure (HB-ACLF) groups. Values are expressed as median ± interquartile range.

To discuss the expression profile of serum suPAR in different states of chronic HBV infection, we included 60 HB-ACLF patients, 38 CHB patients, 33 HBV carriers in immune tolerance phase, and 33 healthy controls. The characteristics of all participants are shown in supplementary Table 1. The results showed CHB patients with hepatic inflammation displayed higher suPAR level than healthy controls (*p* = 0.047), and ACLF patients had the highest level than all other groups (*p* ≤ 0.001). There was no significant difference between IT patients and healthy controls (*p* = 0.806) (Figure 1). To explore potential influence factors, we investigated the correlation of suPAR with various clinical parameters. We found that suPAR levels did not differ according to the gender and age, neither in healthy controls nor HBV-infected patients. There was also no association of suPAR levels with ALT level nor with HBV viral load among IT, CHB, and ACLF patients (data not shown).

Different from western countries where cirrhosis is considered as an essential criterion of ACLF, in Asia-Pacific region, the majority of ACLF is caused by acute severe exacerbation of chronic hepatitis B and cirrhosis is not necessary. A previous study had shown higher suPAR level in cirrhotic than noncirrhotic patients, depending on stage of fibrosis or cirrhosis [11]. To clarify whether this discrepancy affects suPAR expression, we performed a further analysis between HB-ACLF patients with underlying cirrhosis and noncirrhosis. Diagnosis of cirrhosis was confirmed by typical imaging (ultrasound, computed tomographic scan, and magnetic resonance) and/or histological (biopsy) verification of cirrhotic nodules/bridging fibrosis. The characteristics of HB-ACLF patients with underlying cirrhosis and noncirrhosis are shown in supplementary Table 2. Among them, 34 patients were confirmed to have underlying cirrhosis, manifested by older age, lower serum albumin, lower WCC, and platelet counts. However, MELD/CLIF-C OF score and the incidence of complications were similar between both. Unexpectedly, there was no significant difference of serum suPAR suggesting the impact of preexisting cirrhosis on suPAR might be slight in the condition of ACLF (Supplementary Table 2).

### 3.2. Elevated suPAR Level Was Identified as a Strong Predictor of a 90-Day Mortality in HB-ACLF Patients

Subsequently, we investigated whether serum suPAR is associated with the clinical outcome in patients with HB-ACLF. The baseline characteristics of all 60 HB-ACLF patients are shown in Table 1. The median age of patients was 44 (25-67) years, 53 (88.3%) patients were male, and the median MELD was 23 (13-35) at enrollment. The incidence of complications were as follows: clinically proven infection (75%), HE (30.0%), HRS (10%), and GB (8.3%). At the end of the 90-day follow-up period, 15 (25.0%) patients died.

**Figure 2:**
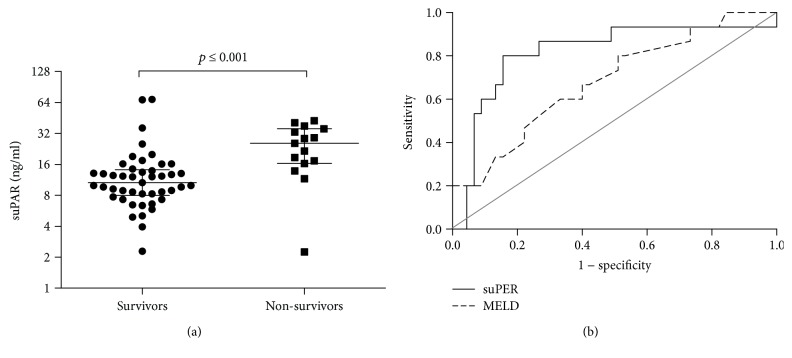
Serum suPAR level at baseline was associated with a 3-month mortality in HB-ACLF patients. (a) Baseline serum suPAR concentration is higher in survivors (*n* = 45) than nonsurvivors (*n* = 15) with hepatitis B-related acute-on-chronic liver failure. Values are expressed as median ± interquartile range. (b) Receiver operating characteristic (ROC) curves of suPAR and MELD score at baseline in HB-ALCF patients. Serum suPAR concentration at baseline has a stronger power for predicting unfavorable outcome as suggested by area under the curve (AUC = 0.816, *p* ≤ 0.001) than MELD score (AUC = 0.682, *p* = 0.036).

Obviously, MELD and CLIF-C OF scores were higher in nonsurvivors than survivors (*p* = 0.029, *p* ≤ 0.001). Nonsurvivors displayed lower serum albumin and more prolonged INR (*p* = 0.023, *p* = 0.043), accompanying with greater frequency of complications including HE, HRS, and GB (Table 1). The results showed that nonsurvivors had higher suPAR level than survivors (*p* ≤ 0.001) (Figure 2(a)), as well as CRP and PCT (*p* ≤ 0.001, Table 1). A strong association was observed among the suPAR, CRP, and PCT levels (Supplementary Figure 1 A-B). Furthermore, there was also a positive correlation among suPAR, INR, and MELD score (Supplementary Figure 1 C-D). Receiver operating characteristic (ROC) curves were generated to access the predicting value of baseline suPAR and MELD score for a 90-day mortality in HB-ACLF patients. Comparing with MELD score (AUC = 0.682, *p* = 0.036), elevated suPAR level was also identified as a strong predictor and more powerful for predicting unfavorable outcome as suggested by area under the curve (AUC = 0.816, *p* ≤ 0.001) (Figure 2(b)).

### 3.3. Longitudinal Dynamics of suPAR between Survivors and Nonsurvivors with HB-ACLF

**Figure 3:**
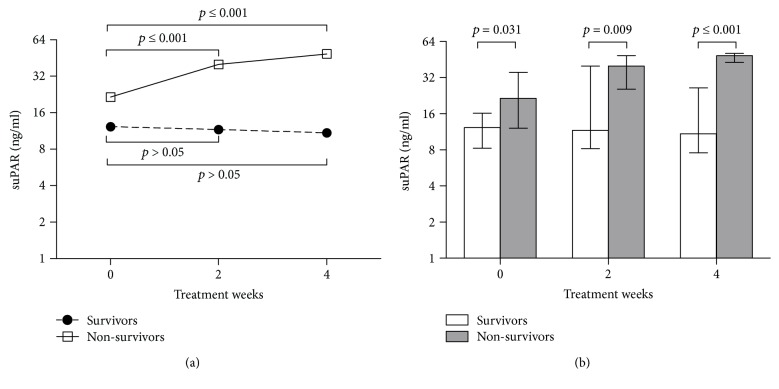
Longitudinal analysis of serum suPAR level between survivors and nonsurvivors in HB-ACLF patients during a 4-week therapy. (a) Temporal dynamics of serum suPAR level in HB-ACLF patients between the survivors and nonsurvivors group. (b) Comparison of suPAR level at individual time between the survivors and nonsurvivors group.

To further demonstrate the clinical impact of suPAR on the outcome, we observed dynamic changes of suPAR during the hospitalization of HB-ACLF. Fifty HB-ACLF patients (including 39 survivors and 11 nonsurvivors) were followed up prospectively, all with available serum at specified time points of baseline, week 2 and week 4 during therapy. Dynamic suPAR expression was differently regulated during a 4-week therapy between the survivors and nonsurvivors. The results exhibited a significant increase of suPAR expression in nonsurvivors during week 0 to week 4 (0 w vs. 2 w: *p* ≤ 0.001, 0 w vs. 4 w: *p* ≤ 0.001), while seemingly descending in survivors (*p* > 0.05, Figure 3(a)). At all indicated time points, serum suPAR level was higher in nonsurvivors than survivors (*p* = 0.031, *p* = 0.009, and *p* ≤ 0.001, Figure 3(b)). A similar tendency of MELD score and INR dynamics was also observed, further indicating the close correlation between suPAR and the severity of HB-ACLF (Supplementary Figure 2 A-B). The increase or decline of suPAR might imply the exacerbation or amelioration of excessive immune activation, eventually leading to immune exhaustion or recovery. The dynamic changes reemphasized the possible relationship between suPAR expression and the prognostic information in HB-ACLF.

### 3.4. A Validation Set for the Predictive Value of suPAR

To validate the predictive value of the suPAR, another cohort from the real-life world was enrolled as an external validation group. The characteristics of all 167 consecutive patients with HB-ACLF are shown in Supplementary Table 3, and 64 (38.3%) patients died at the end of the 90-day follow-up period. The clinical characteristics were basically consistent between the training cohort and validation cohort, except for HBeAg status, HBV-DNA level, and leukocyte counts (Supplementary Table 3). Consistently, no significant difference of serum suPAR was found between HB-ACLF with preexisting cirrhosis and noncirrhosis (data not shown). To explore the factors for predicting the survival of HB-ACLF, we performed the univariate analysis of different clinical variables in the validation set. Similar to the training set, higher MELD/CLIF-C OF score and more severe complications were also found in nonsurvivors than survivors. It was validated that baseline serum suPAR level was higher in nonsurvivors than survivors (*p* ≤ 0.001) (Table 2). A strong association was observed between serum suPAR and a 90-day mortality. In multivariate analysis, baseline suPAR, age, and development of HE were all independent factors for predicting a 90-day mortality of HB-ACLF patient (Supplementary Table 4). Unexpectedly, other inflammatory markers such as WCC, CRP, and PCT were not independent predictors of the outcome.

**Figure 4:**
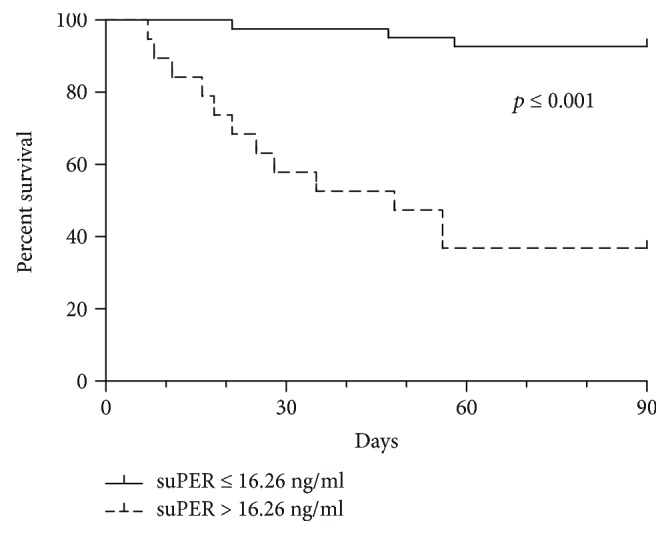
Three-month survival probability curves of hepatitis B-related ACLF patients included in the training set according to suPAR stratification. Survival curves of patients classified according to the stratification of systemic suPAR level with a cut-off of 16.26 ng/ml during hospital stay. *p* ≤ 0.001 is obtained by the log-rank test within the two defined strata.

In the training set, we got the optimal cut-off value of suPAR as 16.26 ng/ml by ROC curve analysis. Survival curves showed that HB-ACLF patients with suPAR > 16.26 ng/ml at baseline had a higher 90-day mortality compared to suPAR ≤ 16.26 ng/ml (*p* ≤ 0.001, Figure 4). To further verify the predictive value of suPAR, all 167 HB-ACLF patients in the external validation group were also classified according to the stratification of systemic suPAR level with a cut-off value of 16.26 ng/ml. Among them, 54 patients had serum suPAR > 16.26 ng/ml, and 39 of these patients died in the hospital or after being discharged from the hospital. By contrast, 88 of the 113 HB-ACLF patients who had serum suPAR ≤ 16.26 ng/ml survived. As shown in Table 3, the cut-off value of serum suPAR > 16.26 ng/ml (60.94% sensitivity and 85.44% specificity) had a positive predictive value (PPV) of 72.22% and a negative predictive value (NPV) of 77.88%. Similar to the training set, survival curves showed that HB-ACLF patients with suPAR > 16.26 ng/ml at baseline had a higher 90-day mortality in the validation cohort (*p* ≤ 0.001, Supplementary Figure 3).

## 4. Discussion

In this study, we investigated the predictive value of suPAR as the immune activation marker in HB-ACLF patients. Our data showed that serum suPAR level was significantly elevated in HB-ACLF patients. Increased serum suPAR correlated with HB-ACLF disease severity and predicted short-term mortality. Nonsurvivors displayed higher suPAR level at enrollment than survivors and progressive elevation during longitudinal course. Serum suPAR on admission was confirmed as a good predictor of HB-ACLF outcome, and the optimal cut-off value was 16.26 ng/ml.

An initial excessive systemic inflammatory response is central to the development of ACLF. In the CANONIC study, ACLF displays key features of SI and its poor outcome is closely associated with exacerbated systemic inflammatory response [2]. Similar opinion was also confirmed in a large cohort of HB-ACLF population in China, emphasizing the putative importance of SI in the pathogenesis [15]. However, most of the biomarkers like WCC and CRP, as well as elevated levels of TNF, IL-6, and IL-18, are nonspecific and not sensitive enough to detect of systemic inflammation early and reliably, especially under noninfectious states. Recently, many potential markers reflecting SI or immune activation have been explored in ACLF. Caspase-cleaved keratin 18 and full-length keratin 18, signifying apoptotic and total cell death, has been found to closely reflect the severity of SI in ACLF [16]. Macrophage activation markers, sCD163 and soluble mannose receptor, are suggested to predict mortality in ACLF [17]. Serum macrophage inflammatory protein 3*α* levels also predict the severity of HB-ACLF [18]. Additionally, other inflammatory mediators, e.g., CXCL10 [19], extracellular histones [20], and DAMP molecular IL-33 [21], have been observed to closely relate with the prognosis of ACLF.

suPAR is an emerging biomarker reflecting immune activation in patients with systemic inflammation or infection [10]. In contrast to many proinflammatory cytokines, suPAR exhibits favorable properties due to its high stability and limited circadian changes in serum samples. It has been proved to be the risk marker in many low-grade inflammation disease such as type 2 diabetes, cardiovascular disease, and cancer and overall mortality even in the general population [22]. Recently, suPAR has been shown in a large multicenter study to be a major predictor on non-AIDS event and death in persons with HIV [23].

Increasing evidences have suggested that systemic suPAR level was elevated in patients with chronic liver diseases, especially associating with progressive liver fibrosis or cirrhosis. Consistent with the aforementioned discussion, we also found higher serum suPAR level in CHB patients than that in heathy controls and HBV carriers in immune tolerance phase. The results indicated the origin of rising suPAR might be related with hepatic inflammation and fibrosis, but not induced by HBV infection itself. Serum suPAR did not correlate with ALT or AST activities, while it has been observed suPAR level is closely correlated with systemic inflammatory cytokines and chemokines including TNF, IL-6, or IL-8 in a previous study [11]. In vitro experiments have proposed that activated monocytes and liver-resident macrophages might be the major source of circulating suPAR in ALF and CLD, which play a role in the pathogenesis of hepatic inflammation and fibrosis. Furthermore, it has been found that activated neutrophils release suPAR in the inflammatory response or infection [24, 25]. This might also contribute to high suPAR expression due to bacterial infection commonly happened in ACLF. The possible dual effect promotes the far higher suPAR expression in ACLF than CHB, while might also dilute the difference of suPAR between preexisting cirrhosis or not in ACLF patients.

Additionally, the importance of suPAR as a potential biomarker in critical illness with systemic inflammation or infection has also garnered a lot of interest. In critically ill patients with SIRS, sepsis, or bacteremia, suPAR is superior in predicting mortality compared to other frequently used biological markers, including CRP, PCT, and sTREM-1. It is worth mentioning that suPAR is closely correlated with liver function in acutely as well as critically ill patients [9, 26]. Systemic inflammation has been considered as an important factor in the development of liver failure. Therefore, it is of interest to elucidate the clinical relevance of suPAR in severe liver disease. Previously, Koch et al. had found that circulating suPAR level was significantly increased in acute liver failure (ALF), independent from the underlying etiology [12]. And decompensated cirrhosis patients also had significantly higher serum suPAR than age- and gender-matched patients with compensated cirrhosis [13]. The significant elevation of serum suPAR in ALF and decompensated cirrhosis suggested it might serve as a promising biomarker correlated with unfavorable clinical outcome. However, only 7 patients were affirmed to be with ACLF and multiorgan failure in Zimmermann et al.'s study [13], limiting the expansibility of suPAR for predicting the survival in ACLF.

Differ from alcoholic or HCV-related cirrhosis in west, the major triggering event is exacerbation of HBV and cirrhosis is not necessary for the development of liver failure in Asia [1, 14, 27]. Furthermore, the clinical presentation of ACLF is different between the East and the West. HB-ACLF was mainly manifested as liver failure and coagulation failure regardless of other multiorgan failure, which is distinct from predominant extrahepatic insults in CANONIC study. Nevertheless, the changes and clinical relevance of circulating suPAR in HB-ACLF keep consistent with decompensated cirrhosis previously reported. It showed that serum suPAR level was significantly elevated in patients with HB-ACLF, correlated with parameters reflecting systemic inflammation (WCC, CRP, and PCT) and severity of ACLF (INR, MELD, and CLIF-C OF score). A positive correlation was observed between serum suPAR and 90-day mortality in HB-ACLF. In the study, we also perform longitudinal measurement of suPAR during the clinical course of HB-ACLF. The gradual increase of suPAR level might be related with the unfavorable prognosis, further verifying the close relevance of suPAR with outcome. It is worthy to note that we demonstrate serum suPAR level > 16.26 ng/ml had a reliable prognostic accuracy for predicting 90-day mortality in HB-ACLF patients through an external validation group. Superior to common inflammatory markers (WCC, CRP, and PCT), suPAR was confirmed as an independent factor for the outcome in ACLF. Thus, we believe that serum suPAR can be considered as an indicator of short-term mortality in HB-ACLF.

There are some limitations in our study. First, it was originated from a single center and made this analysis susceptible to selection bias. Second, it had not differentiated the impact of infectious and noninfectious factors on ACLF, making it confuse to define the value of suPAR. Third, we did not observe the dynamic change of suPAR expression at earlier time points in the first week. As we all known, the initial systemic inflammatory response syndrome due to cytokine storm is closely related with acute insult in ACLF. These cascades of events progress through a “golden window” period of about 7 days, important to early sepsis, organ failure, and survival. Prompt interventions in this “golden window” may improve the outcome of ACLF.

In conclusion, circulating suPAR is increased in patients with HB-ACLF and related to the severity and 90-day mortality. The cut-off value of serum suPAR level > 16.26 ng/ml could be a promising prognostic biomarker for HB-ACLF patients. Further studies in large prospective multicenter settings should be set to evaluate the value of suPAR in HB-ACLF.

## Figures and Tables

**Table 1:** Baseline demographic, clinical, and laboratory characteristics of HB-ACLF survivors and nonsurvivors in the training set.

	Total	Survivors	Nonsurvivors	*p*
Number	60	45	15	
Age (yr)	44 (25-67)	42 (25-63)	51 (31-67)	≤0.001
Sex, male (*n*, %)	53 (88.3)	40 (88.9)	13 (86.7)	0.816
HBeAg positive (*n*, %)	39 (65.0)	28 (62.2)	11 (73.3)	0.435
HBV-DNA (lg copies/ml)	6.93 (2.97-9.02)	7.08 (2.97-9.02)	6.66 (3.63-9.00)	0.335
Serum bilirubin (mg/dl)	18.88 (7.39-36.68)	18.58 (8.12-36.68)	21.43 (7.39-28.40)	0.242
Serum albumin (g/l)	29 (18-38)	30 (18-38)	27 (23-33)	0.023
ALT (U/ml)	644.5 (64-3291)	515 (64-3291)	677.8 (206-1227)	0.389
AST (U/ml)	365.3 (45-2265)	317 (45-2265)	590.9 (126-1231)	0.099
INR	2.21 (1.66-4.60)	2.14 (1.66-3.44)	2.66 (1.73-4.60)	0.043
Baseline sCr (mg/dl)	65 (32.83-108)	64 (36-108)	70 (32.83-99)	0.191
Leukocyte count (10^9^/l)	6.64 (2.69-13.59)	6.65 (2.69-13.59)	6.28 (3.63-12.37)	0.959
Platelet count (10^9^/l)	109 (44-309)	109 (44-309)	105 (49-166)	0.585
MELD score	23 (13-35)	23 (13-29)	25 (20-35)	0.029
CLIF-C OF	9 (7-12)	9 (7-12)	10 (8-12)	0.001
CRP (ng/ml)	6.89 (1.25-67.58)	6.04 (1.31-67.58)	12.71 (1.25-29.50)	≤0.001
PCT (ng/ml)	0.45 (0.12-5.90)	0.41 (0.14-5.90)	0.82 (0.12-2.0)	≤0.001
SuPAR (ng/ml)	12.61 (2.25-68.44)	10.61 (2.29-68.44)	25.68 (2.25-42.61)	≤0.001
Complications (*n*, %)				
Infection	45 (75)	31 (68.9)	14 (93.3)	0.086
Gastrointestinal bleeding	5 (8.3)	0 (0)	5 (33.3)	0.001
Hepatic encephalopathy	18 (30)	9 (20)	9 (60)	0.016
HRS	6 (10)	0 (0)	6 (40)	≤0.001

*n*: number; yr: year; sCr: serum creatinine; MELD: model for end-stage liver disease; CLIF-C OF: chronic liver failure consortium organ failure; CRP: C-reactive protein; PCT: procalcitonin; HRS: hepatorenal syndrome.

**Table 2:** Baseline demographic, clinical, and laboratory characteristics of HB-ACLF survivors and nonsurvivors in the validated set.

	Total	Survivors	Nonsurvivors	*p*
Number	167	103	64	
Age (yr)	43 (19-69)	40 (19-69)	44.5 (24-66)	0.155
Sex, male (*n*, %)	140 (83.8)	86 (83.5)	54 (84.3)	0.881
HBeAg positive (*n*, %)	74 (44.3)	46 (44.7)	28 (43.8)	0.908
HBV-DNA (lg copies/ml)	5.93 (1.15-9.82)	5.82 (1.70-9.82)	6.10 (1.15-9.43)	0.376
Serum bilirubin (mg/dl)	20.19 (6.49-73.01)	17.85 (6.49-73.01)	22.69 (10.08-54.42)	0.001
Serum albumin (g/l)	30 (17-45)	31 (19-45)	29 (17-42)	0.167
ALT (U/ml)	549 (21-4747)	537 (21-4747)	578 (29-3742)	0.603
AST (U/ml)	335 (28-4202)	320 (49-4202)	365 (28-2598)	0.799
INR	2.22 (1.30-14.58)	2.07 (1.30-4.19)	2.95 (1.55-14.58)	≤0.001
Baseline sCr (mg/dl)	67 (33-378)	66 (38-221)	68 (33-378)	0.653
Leukocyte count (10^9^/l)	7.46 (2.39-22.34)	6.87 (2.87-15.6)	8.29 (2.39-22.34)	0.016
Platelet count (10^9^/l)	108 (30-246)	108 (30-211)	111.5 (33-246)	0.824
MELD score	24 (16-50)	23 (16-32)	27.5 (16-50)	≤0.001
CLIF-C OF	10 (6-16)	9 (6-12)	11 (8-16)	≤0.001
CRP (ng/ml)	10.9 (0.65-138.00)	10.5 (2.3-138)	12.08 (0.65-137)	0.979
PCT (ng/ml)	0.63 (0.16-34.99)	0.64 (0.24-2.35)	0.70 (0.16-34.99)	0.184
SuPAR (ng/ml)	10.75 (1.18-121.76)	7.85 (1.18-65.38)	19.8 (1.36-121.76)	≤0.001
Complications (*n*, %)				
Infection	108 (64.7)	57 (55.3)	51 (79.7)	0.001
Gastrointestinal bleeding	18 (10.8)	7 (6.8)	11 (17.2)	0.035
Hepatic encephalopathy	63 (37.7)	14 (13.6)	49 (76.6)	≤0.001
HRS	13 (7.8)	2 (1.9)	11 (17.2)	0.001

*n*: number; yr: year; sCr: serum creatinine; MELD: model for end-stage liver disease; CLIF-C OF: chronic liver failure consortium organ failure; CRP: C-reactive protein; PCT: procalcitonin; HRS: hepatorenal syndrome.

**Table 3:** Diagnostic accuracy of serum suPAR level at admission for predicting 3-month mortality in HB-ACLF patients.

Cut-off point	Total (*n*)	Nonsurvivors (*n*)	Survivors (*n*)	Se (%)	Sp (%)	PPV (%)	NPV (%)	*p*
>16.26 ng/ml	54	39	15	60.93	85.44	72.22	77.88	≤0.001
≤16.26 ng/ml	113	25	88					

*n*: number; Se: sensitivity; Sp: specificity; PPV: positive predictive value; NPV: negative predictive value.

## Data Availability

The data used to support the findings of this study are available from the corresponding authors upon request.

## Abbreviations

ACLF: Acute-on-chronic liver failure
suPAR: Soluble urokinase plasminogen activator receptor
CHB: Chronic hepatitis B
IT: Immune tolerance
HC: Healthy controls
WCC: White cell count
CRP: C-reactive protein
IL: Interleukin
TNF: Tumor necrosis factor
PCT: Procalcitonin
ALF: Acute liver failure
HB-ACLF: Hepatitis B-related ACLF
GB: Gastrointestinal bleeding
HE: Hepatic encephalopathy
HRS: Hepatorenal syndrome
MELD: Model for end-stage liver disease
CLIF-C OF: Chronic liver failure consortium organ failure
ROC: Receiver operating curve
AUC: Area under the curve
PPV: Positive predictive value
NPV: Negative predictive value.

## Supplementary Materials

Supplementary Materials Supplementary Table 1 shows the characteristics of patients in different states of chronic HBV infection, including 60 HB-ACLF patients, 38 CHB patients, 33 HBV carriers in immune tolerance phase, and 33 healthy controls. Supplementary Table 2 compares the baseline demographic, clinical, and laboratory characteristics of HB-ACLF patients between the training and validation cohort. Supplementary Table 3 compares the baseline demographic, clinical, and laboratory characteristics of HB-ACLF with underlying cirrhosis or not in the training set. Supplementary Table 4 shows the independent factors associated with 90-day mortality in the validation set by a multivariate logistic regression analysis. Supplementary Figure 1 displays the correlations of serum suPAR with inflammatory markers, hepatic coagulation function, and MELD score. Supplementary Figure 2 displays longitudinal dynamics of MELD score and INR between survivors and nonsurvivors in HB-ACLF patients during a 4-week therapy. Supplementary Figure 3 displays 90-day survival probability curves of HB-ACLF patients included in the validated cohort according to suPAR stratification by cut-off 16.26 ng/ml at admission.
