# Supplementary material for: Increased Serum Soluble Urokinase Plasminogen Activator Receptor Predicts Short-Term Outcome in Patients with Hepatitis B-Related Acute-on-Chronic Liver Failure
Source: Gastroenterol Res Pract. 2019 May 2;2019:3467690. doi: 10.1155/2019/3467690 (PMC6525912; doi:10.1155/2019/3467690)
Supplement: Supplementary Materials — Supplementary Table 1 shows the characteristics of patients in different states of chronic HBV infection, including 60 HB-ACLF patients, 38 CHB patients, 33 HBV carriers in immune tolerance phase, and 33 healthy controls. Supplementary Table 2 compares the baseline demographic, clinical, and laboratory characteristics of HB-ACLF patients between the training and validation cohort. Supplementary Table 3 compares the baseline demographic, clinical, and laboratory characteristics of HB-ACLF with underlying cirrhosis or not in the training set. Supplementary Table 4 shows the independent factors associated with 90-day mortality in the validation set by a multivariate logistic regression analysis. Supplementary Figure 1 displays the correlations of serum suPAR with inflammatory markers, hepatic coagulation function, and MELD score. Supplementary Figure 2 displays longitudinal dynamics of MELD score and INR between survivors and nonsurvivors in HB-ACLF patients during a 4-week therapy. Supplementary Figure 3 displays 90-day survival probability curves of HB-ACLF patients included in the validated cohort according to suPAR stratification by cut-off 16.26 ng/ml at admission. [file 3467690.f1.pdf]

Supplementary Table 1. The characteristics of patients in different states of chronic HBV infection

	HC	IT	CHB	HB-ACLF
**Number**	33	33	38	60
**Age(yr)**	40(29-52)	29(20-51)	39(20-62)	44(25-67)
**Sex,male(n,%)**	24(72.7)	27(81.8)	30(78.9)	53(88.3)
**HBeAg positive(n,%)**	NA	33(100)	23(60.5)	39(65.0)
**HBV-DNA(lg copies/ml)**	NA	6.75(3.72 -8.30)	6.59(2.70-8.00)	6.93(2.97-9.02)
**Serum bilirubin(mg/dl)**	0.98(0.40-1.32)	1.03(0.30-1.26)	1.08(0.40-10.46)	18.88(7.39-36.68)
**ALT(U/ml)**	23(10-38)	25(9-42)	289.5(51-2339)	644.5(64-3291)
**AST(U/ml)**	19(11-33)	26(9-45)	138.5(33-1258)	365.3(45-2265)

n, number; yr, year; NA, not applicable.

Supplementary Table 2. Baseline demographic, clinical, and laboratory characteristics of HB-ACLF with underlying cirrhosis or not in the training set

	**Total**	**ACLF with LC**	**ACLF without LC**	***p***
**Number**	60	34	26	
**Age(yr)**	44(25-67)	44.5(26-67)	41.5(25-51)	0.026
**Sex,male(n,%)**	53(88.3)	29(85.3)	24(92.3)	0.688
**HBeAg positive(n,%)**	39(65.0)	23(67.6)	16(61.5)	0.623
**HBV-DNA(lg copies/ml)**	6.93(2.97-9.02)	6.56(2.97-9.00)	7.43(3.01-9.02)	0.146
**Serum bilirubin(mg/dl)**	18.88(7.39-36.68)	17.721(8.12-31.17)	19.13(7.39-36.68)	0.829
**Serum albumin(g/L)**	29(18-38)	28(18-35)	31(25-38)	0.001
**ALT(U/ml)**	644.5(64-3291)	539.5(64-2554)	683.7(277-3291)	0.059
**AST(U/ml)**	365.3(45-2265)	293.5(45-1458)	501.5(107-2265)	0.065
**INR**	2.21(1.66-4.60)	2.11(1.66-4.09)	2.61(1.73-4.60)	0.012
**Baseline sCr(mg/dl)**	65(32.83-108)	64(36-108)	70(32.83-99)	0.328
**Leukocyte count(10^9/L)**	6.64(2.69-13.59)	6.02(2.69-11.89)	7.37(4.71-13.59)	0.008
**Platelet count(10^9/L)**	109(44-309)	94(44-144)	142(115-309)	≤0.001
**MELD score**	23(13-35)	23 (13-33)	24.5(21-35)	0.093
**CLIF-OF**	9(7-12)	9(7-11)	10(8-12)	0.113
**SuPAR(ng/ml)**	12,61(2.25-68.44)	13.04(2.25-68.44)	11.04(2.29-68.05)	0.416
**Complications(n, %)**				
**Infection**	45(75)	27(79.4)	18(69.2)	0.367
**Gastrointestinal bleeding**	5(8.3)	3(8.8)	2(7.7)	1.000
**Hepatic encephalopathy**	18(30)	10(29.4)	8(30.8)	0.909
**HRS**	6(10)	3(8.8)	3(11.5)	1.000

n, number; yr, year; sCr, serum creatinine; MELD, Model for End Stage Liver Disease; CLIF-C OF, Chronic Liver Failure Consortium Organ Failure; CRP, C reactive protein; PCT, procalcitonin; HRS, hepatorenal syndrome.

Supplementary Table 3. Baseline demographic, clinical, and laboratory characteristics of HB-ACLF patients between the training and validation cohort

	Training cohort	Validation cohort	*p*
**Number**	60	167	
**Age(yr)**	44(25-67)	43(19-69)	0.900
**Sex,male(n,%)**	53(88.3)	140(83.8)	0.402
**HBeAg positive(n,%)**	39(65.0)	74(44.3)	0.006
**HBV-DNA(lg copies/ml)**	6.93(2.97-9.02)	5.93(1.15-9.82)	0.005
**Serum bilirubin(mg/dl)**	18.24(7.39-35.44)	20.19(6.49-73.01)	0.350
**Serum albumin(g/L)**	29(18-38)	30(17-45)	0.083
**ALT(U/ml)**	644.5(64-3291)	549(21-4747)	0.546
**AST(U/ml)**	365.3(45-2265)	335(28-4202)	0.557
**INR**	2.21(1.66-4.60)	2.22(1.30-14.58)	0.943
**Baseline sCr(mg/dl)**	65(32.83-108)	67(33-378)	0.066
**Leukocyte count(10^9/L)**	6.64(2.69-13.59)	7.46(2.39-22.34)	0.025
**Platelet count(10^9/L)**	109(44-309)	108(30-246)	0.517
**MELD score**	23(13-35)	24(16-50)	0.063
**CLIF-OF**	9(7-12)	10(6-16)	0.098
**SuPAR(ng/ml)**	12,61(2.25-68.44)	10.75(1.18-121.76)	0.116
**Complications(n, %)**			
**Infection**	45(75)	108(64.7)	0.143
**Gastrointestinal bleeding**	5(8.3)	18(10.8)	0.590
**Hepatic encephalopathy**	18(30)	63(37.7)	0.284
**HRS**	6(10)	13(7.8)	0.595

n, number; yr, year; sCr, serum creatinine; MELD, Model for End Stage Liver Disease; CLIF-C OF, Chronic Liver Failure Consortium Organ Failure; suPAR, soluble urokinase plasminogen activator receptor; CRP, C reactive protein; PCT, procalcitonin; HRS, hepatorenal syndrome.

Supplementary Table 4. Independent factors associated with 90-day mortality in the validation set by a multivariate Logistic Regression Analysis

Variable	OR	95%CI	*P* value
**Age**	1.07	1.01-1.13	0.030
**Hepatic encephalopathy**	2.06	1.08-4.24	0.005
**suPAR**	1.12	1.05-1.19	0.001

suPAR, soluble urokinase plasminogen activator receptor.

Supplementary Figure Legends

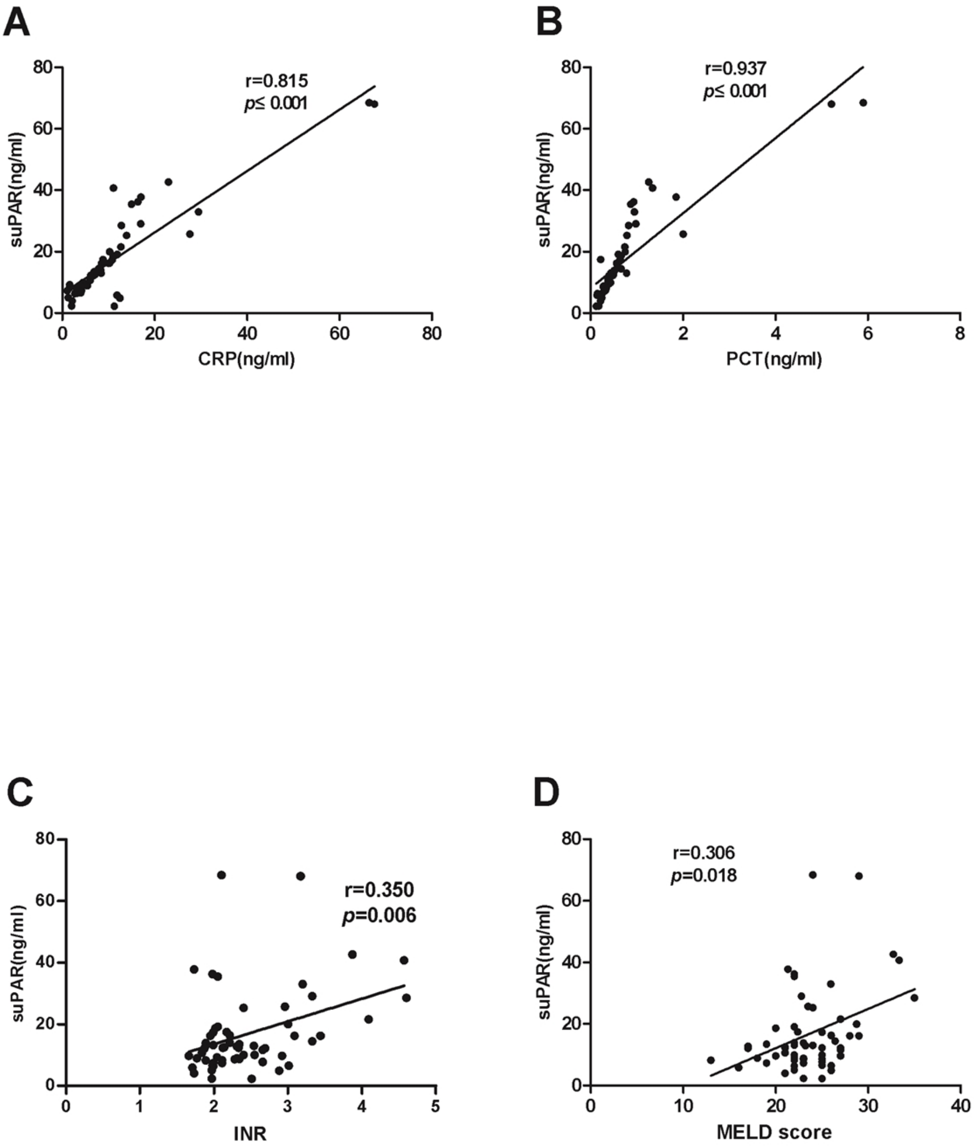

Suppl. Fig. 1: The correlations of suPAR with inflammatory markers, hepatic coagulation function, and MELD score. Serum suPAR level positively correlated with **(A)** systemic CRP level, and **(B)** systemic PCT level , and **(C)** INR value and **(D)** MELD score. Linear correlations with 95% confidence intervals are shown. Pearson correlation coefficients (*r*) and *p* values are given in each panel.

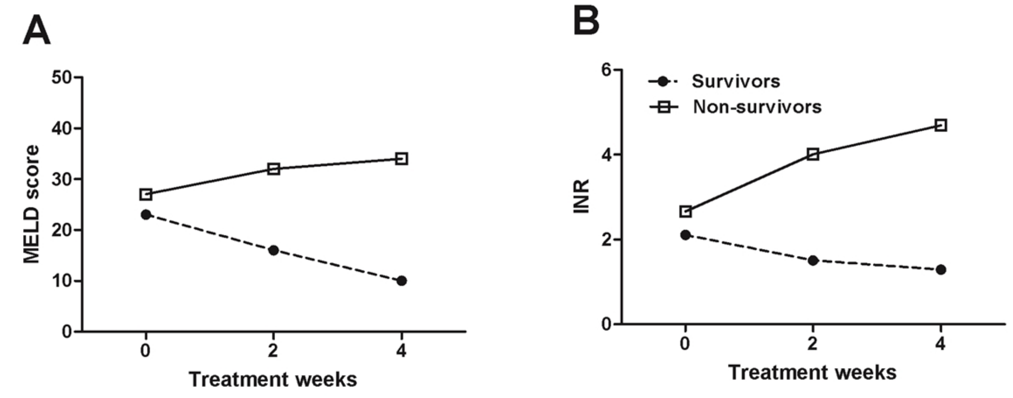

Suppl. Fig. 2: Longitudinal analysis of MELD score, INR between survivors and non-survivors in HB-ACLF patients during 4 weeks therapy. (A) Temporal dynamics of MELD score in HB-ACLF patients between survivors and non-survivors group. **(B)** Temporal dynamics of INR in HB-ACLF patients between survivors and non-survivors group.

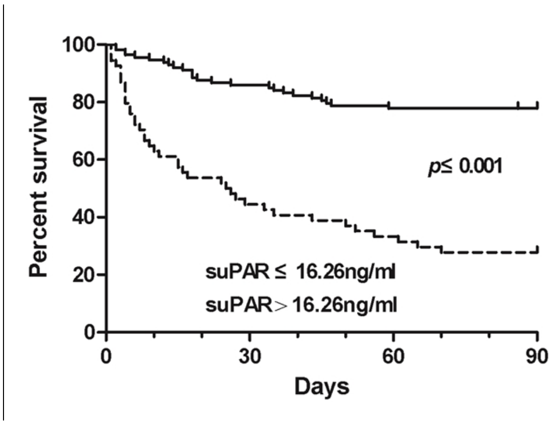

Suppl. Fig. 3: Three-month survival probability curves of hepatitis B-related ACLF patients included in the validate cohort according to suPAR stratification. Survival curves of patients classified according to the stratification of systemic suPAR level with cut-off 16.26ng/ml at admission. *p*≤0.001 is obtained by the log-rank test within the two defined strata.
